# Back Pain Related with Age, Anthropometric Variables, Sagittal Spinal Curvatures, Hamstring Extensibility, Physical Activity and Health Related Quality of Life in Male and Female High School Students

**DOI:** 10.3390/ijerph17197293

**Published:** 2020-10-06

**Authors:** Noelia González-Gálvez, Raquel Vaquero-Cristóbal, Abraham López-Vivancos, Mario Albaladejo-Saura, Pablo Jorge Marcos-Pardo

**Affiliations:** 1 Faculty of Sports, Universidad Católica San Antonio de Murcia, Murcia, Campus de los Jerónimos, 30107 Guadalupe, Murcia, Spain; ngonzalez@ucam.edu (N.G.-G.); alvivancos@ucam.edu (A.L.-V.); pmarcos@ucam.edu (P.J.M.-P.); 2 Kinanthropometry International Chair, Universidad Católica San Antonio de Murcia, Murcia, Campus de los Jerónimos, 30107 Guadalupe, Murcia, Spain; mdalbaladejosaura@ucam.edu

**Keywords:** low back pain, physical activity, postural habits, quality of life, secondary school, spine

## Abstract

Spinal pain (SP) is widely extended among adolescents. The origin of SP can be multifactorial; thus, the present study aimed to estimate the prevalence and risk of SP in high school students and to determine the differences in sagittal spinal curvatures and pelvic tilt, hamstring extensibility, age, anthropometric variables and healthy lifestyle habits dependent on SP between sexes. Two hundred seventy-three teenagers took part in this cross-sectional study. Age, sagittal spinal curvatures, hamstring extensibility, physical activity, sedentary lifestyle, anthropometric variables and health related quality of life (HRQL) were recorded. SP was reported by 16.12% of adolescents. Differences were observed in the HRQL according to SP (*p* < 0.05). Participants without SP were less sedentary (22.12%) and younger (13.10 years old) than participants with SP (40.91% and 13.66, respectively) (*p* < 0.05). A logistic regression model showed that both variables were significantly collinear (VIF = 1.01; Durbin-Watson = 2.10). Subjects with low back pain (LBP) had a higher weight, body max index, and hip girth than subjects without pain (*p* < 0.05). A misalignment in the lumbar spine was associated with LBP for males (Cramer’s V = 0.204, *p* = 0.022). In conclusion, adolescents with SP were older and had a lower HRQL in all dimensions. SP could be predicted according to age and sedentary habits.

## 1. Introduction

Low back pain (LBP), defined as pain experienced in the region of the lumbar and sacrum spine, between the boundaries of L1-S5 and across the posterior aspect of the lower back [1,2], is the leading cause of disability in the world [3] and is found within the top ten types of pain among adolescents [4]. This topic is also important because associations between adolescent and adult LBP has been observed [5,6]. Although most of the studies in adults have analyzed LBP, evidence suggests that the importance of cervical pain (CP), defined as pain experienced in the region of the cervical spine, between the boundaries of C1-C7 and across the posterior aspect of the neck [2,7], and thoracic spine pain (TSP), defined as pain experience in the region of the thoracic spine, between the boundaries of T1-T12 and across the posterior aspect of the trunk [7], should also be analyzed during adolescence because the incidence and prevalence of pain in different areas of the spine are high at this growth stage [1,2,7,8,9]. Therefore, spinal pain (SP) as a global concept that encompasses CP, TSP and LBP, defined as pain perception in the neck or back that could be influenced by spine bones, ligaments, muscles and tendons as active and passive structures [10] but also by more central structures [11,12], could be the key during adolescence [9].

The introduction of spinal pain education programs into the curriculum during childhood and adolescence can be a favorable context for improving knowledge on back health in order to reduce the incidence of SP over the student’s lifetime [13]. Although several authors have developed intervention programs about SP prevention and back health care in elementary school [14,15,16], the incidence of SP increases during the period of growth [17], so interventions in high school are also needed. Previous studies have found that high school students have a low level of specific knowledge about back care and its relationship with health, even those with SP [13]. Interventions based on improving knowledge about back care have found excellent results [18]. However, although these kinds of programs can be effective in increasing knowledge about back care up to adulthood, they do not change back care behavior in the long term [16].

Another option is to carry out specific exercise programs in high school to improve factors that could be related with SP [19,20,21]. Along this line, SP has been linked to spine misalignments [7,22]; shortness in hamstring extensibility [23]; body height, body mass index (BMI), waist and hip girths [23,24]; or sedentary behaviors during leisure time [24,25] in childhood and adolescence. However, SP is characterized by a multifactorial condition which involves several risk factors such as sociodemographic, physiological or psychosocial factors [2], and most of the prior studies focused on a period of growth only used a one-factor approach.

This multifactorial focus is essential in adolescence because most of the biomechanical and physiological parameters rapidly change during this period of growth as a consequence of the effects of sexual dimorphism on the body structure, morphology and physiology [26,27,28]. Furthermore, some changes are also found in their habits. For example, adolescents prefer to spend their leisure time in sedentary activities with friends as opposed to performing a physical activity (PA) [29]. As a result, the importance of these factors on SP and their interinfluence can be different depending on sex [23,24,30] and age [31]. As a consequence, it is necessary to analyze the prevalence of SP risk SP depending of these variables from a global point of view and including pain in the different areas of the spine to understand how to focus interventions in high school and what could be the most influencing factors for each age and sex.

Therefore, the aims of this study were: (a) to estimate the prevalence and risk of SP in high school students and (b) to determine the differences in age, sagittal spinal curvatures and pelvic tilt, hamstring extensibility, anthropometry variables, PA level, leisure-time sedentary behaviors and health related quality of life (HRQL) dependent on SP between sexes. The hypotheses were the following: (a) highschool students suffer SP in different areas, with LBP being the most common; (b) SP incidence increases with age during adolescence; and (c) the association between SP and biomechanical, physiological, psychological and sociological factors depend on sex. If these hypotheses are supported, it would indicate that educational programs on SP implemented in high school will have to include contents about CP, TSP and LBP, and utilize different approaches depending on the sex and age of the students.

## 2. Materials and Methods

### 2.1. Design and Setting

The present study was a cross-sectional study conducted at a high school and sports science laboratory. The trial design was registered with ClinicalTrial.gov (identifier: NCT03831867). All parents or tutors signed an informed consent approved by the "Scientific and Ethical Committee" of the institution (code: TC4/17), and the study was conducted in accordance with the Declaration of Helsinki. The cross-sectional study design followed the STROBE Statement. The data associated with the present word are available in the INVESALUD repository, from the institution’s foundation. It is also available from the corresponding author at: https://drive.google.com/file/d/15_0peSh3ypNbJHfPT5bkW8EpvWqaotee/view?usp=sharing.

### 2.2. Participants

Two hundred seventy-three teenagers (male: MS = 134, 49.08%; female: FS = 139, 50.92%) aged between 12 and 17 years old (average age: 13.19 ± 1.2 years old) participated in this study. They were all students in a high school from the Region of Murcia (Spain). The criteria for inclusion were the following: (a) to be free of musculoskeletal, neurological, cardiac, metabolic or rheumatic illnesses and (b) to be a student in a high school. The exclusion criterion was to be employed in any kind of work.

The calculations utilized to establish the sample size were performed using Rstudio 3.15.0 software (RStudio, Northern Ave, Boston, MA, USA). The significance level was set at α = 0.01. According to the standard deviation established for the thoracic sagittal curvature of the spine measured with the Spinal Mouse in a previous study [32] and an estimated error of 2 degrees, a valid sample size for a confidence interval of 99% was 142.38. A total of 273 students completed the trial, providing a power of 99% if found between and within a variance of 1.44°. A power of 95% and an estimated error of 2.86 degrees was found in connection with the group of adolescents with LBP (*n* = 40).

### 2.3. Outcome Measures

All the measurements were performed by the same researchers in a single session between the h of 10:00 and 14:00. No warm-up or stretching exercises were performed by the participants before the test measurements. The participants were examined barefoot. The laboratory temperature was standardized at 24 °C. There was a 5-min rest between measurements. Prior to the examination, and in order to establish the reliability of the examiner, a double-blind study was performed with 30 subjects, obtaining an intraclass correlation coefficient higher than 95%.

To assess the sagittal spinal curvatures and pelvic tilt, the Spinal Mouse System (Idiag, Fehraltdorf, Switzerland) was utilized. The Spinal Mouse is an electronic device connected via Bluetooth to a computer, and was used to measure the sagittal spinal range, by measuring the angle of the thoracic curve, lumbar curve and pelvic tilt in standing position, in a non-invasive way, following the Muyor et al. protocols [32]. It is the most-commonly known and used instrument, and it has a high validity, intrarater reliability (ICCs: 0.61–0.96) and interrater reliability (ICCs: 0.70–0.93) [33].

For the thoracic curve, values below 20° were considered as hypokyphosis, values between 20 and 45° as neutral kyphosis and values higher than 46° as hyperkyphosis. For the lumbar curvature, values higher than −20° were considered as hypolordosis curves, values between −20 and −40° as neutral lordosis and values lower than −40° as hyperlordosis [34]. This classification for spinal curvatures has been used in several studies with adolescents [22,35].

The sit-and-reach (SR) test was used to evaluate hamstring flexibility following the Muyor et al. protocols [36]. To evaluate the pelvic tilt, the sagittal spinal curvature was assessed in this position at the start of the measurement at the T1 mark in flexion with the Spinal Mouse System. Regarding the pelvic tilt, 0° indicated a neutral position, negative values indicated pelvic retroversion, and positive values indicated pelvic anteversion [36].

The passive straight leg raise (PSLR) tests were performed according to Muyor et al. [36]. Both legs were measured and the mean was calculated afterwards. 

The Back Pain Adolescent Survey utilized to discover the prevalence of SP, CP, TSP or LBP; PA; and sedentary behaviors during leisure-time was designed and validated by Martínez-Crespo et al. [37], with a kappa coefficient > 0.75. SP, CP, TSP, LBP in the last year and SP in last week were asked about. The practice of PA, the h of practice and the h of sedentary time were assessed. A new variable was calculated using a combined number of h of practice PA and number of h spend in sedentary time. It was considered sedentary when spending more than two h per day, following the recommendations from the Physical Activity Guidelines Advisory Committee [38], and it was considered active when spending more than 6 h per week in physical activities [39]. As a result, four categories from this variable were possible: Not sedentary and Active; Not sedentary and Not Active; Sedentary and Active; and Sedentary and Not Active.

A SECA 762 scale (SECA, Germany) was used to measure body mass, a GPM anthropometer (Siber-Hegner, Zurich, Switzerland) was used to measure stretch stature, and a Lufkin W606PM measuring tape (Lufkin, EE.UU.) to measure waist and hip girths. Afterwards, BMI (kg/m^2^) was calculated as body mass (kg)/stretch stature (m)^2^ [40], while waist-to-hip ratio was calculated as the waist measurement divided by the hip measurement (W/H). This last was used as an indirect marker of intra-abdominal obesity [41]. The recommendations from Mederico et al. [42] were followed for the measurements.

HRQL was assessed using the questionnaire KIDSCREEN-27 [43], adapted and validated to Spanish, and showing a Cronbach’s alpha coefficient higher than 0.70 in all its dimensions [44]. This questionnaire, composed of 37 questions, covers four basic dimensions: physical well-being, psychological well-being, autonomy and relationship with parents and social and peer support. It utilizes a Likert scale for scoring. It is possible to obtain individual scores, a dimension score and a global index of HRQL.

### 2.4. Statistical Analysis

After analyzing the normality of the variables with a Kolmogorov–Smirnov test, a descriptive analysis was performed for the quantitative variables (means and standard deviations) and qualitative variables (counts and percentages). Unpaired *t*-tests (continuous variables) evaluated differences between two groups (MS/FS, with SP/without SP). The size effect was calculated, defined as low: r = 0.10; moderate: r = 0.30, high: r = 0.50; very high: r = 0.70 [45]. X^2^ analyses (categorical variables) were used to analyze differences between groups. The statistic Cramer´s V was utilized for the post hoc comparison of 2 × 2 tables, and the statistic contingency coefficient was used for 2 × n tables, showing the value of the statistic and the *p* value. The maximum expected value was 0.707; a low association was indicated if r ˂ 0.3; a moderate association if the r value was between 0.3 and 0.5 and a high association if r > 0.5. Stepwise, multiple logistic regression models were used to predict SP according to age and spending more than 2 h of leisure-time sedentary behaviors. The analysis reported two models: Model 1 for age, and Model 2 for age and 2 h of leisure-time spent in sedentary behaviors. The statistical analysis was performed using the statistical package SPSS 21.0 for Windows (IBM Corp., Armonk, New York, NY, USA). An error of *p* ≤ 0.05 was established.

## 3. Results

Table 1 shows the differences between sexes of the sagittal spine curvatures and pelvic tilt in standing position, hamstring extensibility, age and anthropometric variables. A significant difference between MS and FS was found for the reported degrees of the thoracic curvature, lumbar curvature and pelvic tilt, height, waist girth and waist-to-hip ratio (*p* < 0.05).

Table 2 shows the differences between sexes of the percentages of curvature classification, PA, leisure-time sedentary behaviors and SP. It was found that the majority of the participants had neutral curvatures, practiced PA between 1 and 5 h per week, their leisure-time sedentary behaviors lasted less than two h per day and did not suffer SP. The FS had greater hyperkyphosis and hyperlordosis, practiced less PA and for a lower number of h than the MS (*p* ˂ 0.05). It was also discovered that 16.12% of adolescents reported SP, with LBP being the most prevalent (10.34%). No differences between sexes for SP and leisure-time sedentary behaviors were found.

Table 3 shows the analysis of the different variables according to SP and according to SP categorized according to sex. Significant differences were observed in the dimensions of quality of life according to SP. Subjects with SP were older than those without SP, being significant for FS. In addition, participants with SP obtained lower scores in all dimensions of quality of life than subjects without pain, although only the difference for FS was significant. Separated by CP, TSP or LBP, subjects with LBP had a significantly higher weight (59.58 vs 53.54 kg; *p* = 0.018), BMI (22.04 vs 20.63 kg/m^2^; *p* = 0.037) and hip girth (94.16 vs 90.12 cm; *p* = 0.024) than subjects who did not report pain. No differences were found in the other variables and in CP or TSP.

Table 4 shows associations between the presence of SP and percentages of curvature classification, PA, and leisure-time sedentary behaviors. It was found that the sedentary time of participants without SP was lower than the adolescents with SP in the general sample and FS. Non-significant differences were found for MS or the relationship of SP with the other variables. Separating by CP, TSP or LBP, an association between having a misalignment in the lumbar spine (hypolordosis or hyperlordosis) and LBP for MS (Cramer’s V = 0.204, *p* = 0.022) was observed. A significantly higher percent of boys with LBP had a misalignment in the lumbar curvature (46.15%) than boys without SP (18.58%). Non-significant differences were found for the rest of analyses.

Table 5 shows the results of the new variable calculated, which combined PA and sedentary behaviors. The adolescents considered as sedentary and not active (more than 2 h of sedentary behaviors per day and less than 7 h of PA per week) had the highest percentage of SP in the general sample, MS and FS, being significant for the general sample (contingency coefficient = 0.195; *p* = 0.014) and FS (contingency coefficient = 0.275; *p* = 0.011).

Based on these findings, age and more/less than 2 h spent in leisure-time sedentary behaviors were considered for inclusion into the logistic regression model. Both variables were significantly collinear (VIF = 1.01; Durbin-Watson = 2.10) (Table 6).

## 4. Discussion

To date, none of the studies found in the literature review have analyzed the incidence of SP in different areas of the back in the same sample of adolescents. The first aim of the present study was to estimate the prevalence and risk of SP in high school students. It was found that 16.12% of the students suffered SP, with a prevalence of 2.30% for CP, 5.75% for TSP and 10.34% for LBP, which agrees with hypothesis (a), with a similar incidence according to sex. Percentages of incidence in the present study for SP, TSP and LBP were within the range shown by recent systematic reviews [1,2,8]. Small differences between the percentages of the different studies could be due to differences in the SP, CP, TSP and LBP definitions and methodology used to collect the incidence of pain [24]. According to these data, it is necessary to include intervention programs into the educational context to decrease the percentage of adolescents affected by SP and to decrease the pain of those who suffer from SP [14,15,16,18]. Furthermore, educational programs should not only focus on LBP, as it has frequently occurred [14,15,16,18,19], but should also include knowledge about SP and CP, and the interventions necessary to decrease the incidence of these pathologies.

Another important finding was that the prevalence of SP increased with chronological age, according with hypothesis (b). A similar evolution has been found in previous studies [24,31,46]. This finding has important implications for the introduction of SP education programs in secondary education. Due to the low incidence in the youngest students, back health programs in the first academic years could be focused on improving the knowledge about back care and its relation with SP, life habits, health and quality of life, following the guidelines of previous studies, which have demonstrated their effectiveness [13]. It is possible that this will not incite behavioral changes, but some studies have found that knowledge is needed before a positive change starts [16,47]. As the incidence of SP increases during adolescence, physical interventions during the last years of high school could be an interesting tool for improving back health.

Furthermore, one interesting finding was that the relationship between age and SP was stronger in FS that in MS, although in general no differences were found in the prevalence of pain reported by the adolescents according to sex. A possible explanation for this may be that menarche is the most predictive demographic or medical history factor associated with LBP according to previous data [31]. FS hormones, particularly estrogens, increase after menarche, inducing changes in the girls’ bodies. For example, hips widen during puberty and have an influence on lower-limb alignment. This factor has been correlated with a wider support base, increased static and dynamic knee abduction angles and external moments during walking and more valgus knee in static and dynamic conditions [48], factors that are highly related with SP [49]. Another possible explanation for this is that FS hormone levels are related with increased lumbar spine mobility [50], which is associated with trunk muscle fatigue and excessive spinal loads [51], factors that are also associated with SP [52]. According to these data, it could be inferred that education programs may need a different orientation depending on the student’s sex in the last years of high school.

Quality of life is used as a health outcome to evaluate the physical, social and mental functioning in adolescents [53]. An important and clinically-relevant finding of the present study was that there were differences between SP and no SP groups related to the health-related quality of life in the total sample and FS. In fact, together with age, these were the unique variables included in the logistic regression model to predict SP. Some studies have shown that SP is associated with poorer quality of life in adolescents [54,55,56,57] but few of them have analyzed differences between the sexes [54]. This previous study reported that the association between LBP and lower quality of life was higher in girls [54], which corroborates the present findings and hypothesis (c). Higher associations in girls may be the result of the menstrual cycle due to the menstrual cycle-induced LBP in more than half of FS high school students [58], and it is also associated with frequent complaints and pain [59], factors that affect quality of life [54]. Thus, the menstrual cycle could be the reason for the relationship between both factors. Along this line, some exercise program based on stretching and/or lumbo-pelvic exercises have demonstrated reductions in LBP and improvements in the physiological wellness of women [60,61]. Another possible explanation is that as a consequence of the roles fixed by education and society, boys have become more reticent about showing their symptoms and feelings [62]. Thus, stretching and/or lumbo-pelvic exercises could become interesting topics for exercise intervention programs with FS students, while an increase in the knowledge about the relationship between LBP and quality of life could be interesting for MS students.

One interesting finding of the present study is that a higher percentage of adolescents with SP spent more than 2 h of their leisure-time in sedentary behaviors than the no SP group. Previous studies also found a correlation between LBP and the time spent in sedentary behaviors such as watching TV, in adolescents [62,63], and similar results have been found in the general population [25]. This result may be explained by the fact that a prolonged seated position can result in viscoelastic deformation of passive tissues in the posterior trunk. If it occurs, the activation of trunk muscles is increased [64], and it is when these muscles are fatigued that the incidence of back disorders increases [51] as, for example, LBP [65]. However, no previous study has investigated differences between sexes in the relationship between SP and leisure-time sedentary behaviors. The results of the present study showed that there was a relationship between these factors only in FS, which agrees with hypothesis (c). Trunk muscle resistance and stability capacity could be key aspects for preventing the association between sedentary behaviors and back disorders [66,67]. Previous studies have found that women showed lower trunk flexor and extensor strength and endurance, and lower trunk balance [68,69], so it could be interesting to include specific exercises to improve these factors in the interventions carried out with girls in high school. However, other non-mechanical inputs, linked to the International Classification of Functioning, Disability and Health, as nociceptive, nervous system dysfunction, comorbidities, cognitive-emotional and contextual drivers can cause pain and/or disability in LBP [12,70,71], so global interventions including mechanical and non-mechanical factors may be carried out to improve both functioning and contextual parameters [11,72,73].

No significant differences in the practice of PA and hours of practice were found between the SP and no SP groups. It seems possible that these results were due to the fact that a differentiation between sports was not performed. Along this line, the systematic practice of some sports or activities decreases the risk of suffering SP, especially when the sport increases trunk muscle resistance, trunk stability, extensibility and the spine is in neutral positions; but the practice of other sports characterized by hopping, jogging or stepping, with flexion or extension trunk positions, or by unilateral actions especially if there are repetitive motions, can increase it. This could be, for example, the case of volleyball, tennis, cycling or rowing [24,74,75,76]. The risk increases with training volume, higher physical loads, repetitive mechanical strain and dynamic extreme body positions [76]. It could be important to include knowledge about beneficial sports and harmful sports for SP in the education programs that are developed in high school, because educational programs do not usually include this information.

No previous study has investigated the combination of sedentary behaviors in leisure-time and PA related with SP incidence. In the present study, students that spent more than 2 h of their leisure-time in sedentary behaviors and did not practice PA showed a higher SP incidence than other groups. PA is not only associated with improvement in mechanical factors associated with SP [19,77], but also with non-mechanical factors [12,70,71] as, for example, brain development [78], central mechanism adaptations [79] or immune system functions [80]. Furthermore, in general, not differences were observed between the group of students that showed sedentary behaviors but practiced PA; the group of students that did not show sedentary behaviors but did not practice PA, and group of students that did not show sedentary behaviors and practiced PA. It can thus be suggested that the presence of at least one of these two factors can induce a significant reduction in the incidence of SP, and it could be an interesting guideline to follow in back educational programs. Despite these promising results, many questions still remain.

Another important finding was that students with LBP showed higher body mass, BMI and waist girth than the no LBP group. Previous studies have found a relationship between anthropometric variables and LBP [23,24]. Associations between anthropometry variables and LBP can be a consequence of the extra load on the spine induced by the increases in weight [23]. Based on these findings, educational programs which induce changes in PA and nutritional habits could be effective for reducing LBP incidence. Innovative teaching methods could be excellent tools for achieving this [81]. However, due to the influence of the sex hormones in these factors and the lack of longitudinal studies, future studies with a longitudinal design addressing the relations between LBP and anthropometric variables that include sex hormone values and data about the menstrual cycle are therefore recommended.

In this study, spine curvatures were not related with SP, except when lumbar misalignment and LBP were analyzed in MS. In fact, misalignment was not included in the logistic regression model. Similar results have been found in previous studies, which explained the results by studying the association of spine posture as a single variable [22,82,83]. Furthermore, most of the participants showed neutral curvatures, which can influence the relationship with SP. In this sense, students with a musculoskeletal illness were excluded in the present study, so it could be possible that results could be different when students with a pathology in the sagittal plane of the spine are analyzed [22,35]. This is an important issue for future research.

No differences were found in hamstring extensibility between the SP and no SP groups either. While a study found that girls without LBP had a higher hamstring extensibility than the group with LBP, no differences were found in boys [84]. Another study found that the percentage of boys with hamstring shortness in the LBP group was higher than in the no LBP group, although no differences were found in girls [23]. Lastly, another study did not find a relationship between hamstring extensibility and LBP [24]. Further studies, which analyze the relationships between the spine, hamstring extensibility and SP, and the influence of some aspects such as musculoskeletal, neuroplasticity and biomechanics characteristics during puberty related with sex hormone levels throughout adolescence and depending on sex will need to be undertaken.

Some limitations should be acknowledged. The main limitation was that the assessment of pain did not include information about its duration or severity. A second limitation was that the results provided by this research study should be viewed with caution, as the sample selection does not allow them to be generalized. Furthermore, differences in the approaches utilized to collect data on SP, CP, TSP and LBP prevalence between studies made the comparison of the findings more complex. Lastly, sex hormone levels and information about the menstrual cycle were not measures, although they can influence the relationship between factors, especially during adolescence.

## 5. Conclusions

Of the total sample, 16.12% of the adolescents reported spinal pain, with the most prevalent pain being lumbar pain (10.34%), followed by thoracic spine pain (5.75%) and cervical pain (2.3%), without differences found between both sexes. There was no association between spinal pain and the sex of the participants, the level of PA practiced, height, hip girth or waist-to-hip ratio. Adolescents with spinal pain were older, had a lower quality of life in all dimensions and spent more than 2 h of leisure-time on sedentary behaviors per day. Adolescents with LBP were heavier and had a greater BMI and hip girth than subjects who did not report pain. MS showed an association between LBP and misalignment in the lumbar spine.

## Figures and Tables

**Table 1:** Mean values (±SD) of curvatures, hamstring extensibility and anthropometric variables and differences between sexes.

	Total 100% (*n* = 273)	MS 49.08% (*n* = 134)	FS 50.92% (*n* = 139)	Diff. MS-FS	95%CI	*p*	Effect Size d
Thoracic curvature in standing (°)	35.3 ± 11.4	32.1 ± 10.8	38.4 ± 11.1	−6.3	−8.9; −3.7	0.000	0.57
Lumbar curvature in standing (°)	−35.3 ± 7.2	−33.4 ± 7.3	−37.0 ± 6.6	3.6	2.0; 5.3	0.000	0.52
Pelvic tilt in standing (°)	25.0 ± 7.3	26.0 ± 8.0	24.1 ± 6.4	1.9	0.2; 3.6	0.033	0.24
Distance in SR (cm)	−8.7 ± 9.4	−12.8 ± 8.1	−4.7 ± 8.7	−8.1	−19.1; −6.1	0.000	0.97
Pelvic tilt in SR (°)	−13.9 ± 9.4	−20.4 ± 9.6	−7.7 ± 12.9	−12.7	−15.5; 10.0	0.000	1.12
PSLR (°)	81.8 ± 14.4	74.8 ± 9.2	88.6 ± 15.3	−13.8	−16.8; −10.8	0.000	1.09
Age	13.2 ± 1.2	13.3 ± 1.3	13.1 ± 1.2	0.2	−0.1; 0.6	0.099	0.20
Body mass (kg)	54.2 ± 12.1	55.5 ± 13.0	52.9 ± 11.2	2.6	−0.3; 5.5	0.078	0.21
Height (cm)	161.3 ± 9.4	163.2 ± 10.8	159.6 ± 7.3	3.7	1.5; 5.9	0.001	0.40
BMI (kg/m^2^)	20.7 ± 3.8	20.7 ± 3.9	20.7 ± 3.8	−0.02	−0.9; 0.9	0.974	0.00
Waist girth (cm)	72.3 ± 9.9	74.9 ± 10.5	69.9 ± 8.6	5.0	2.8; 7.3	0.000	0.53
Hip girth (cm)	90.3 ± 8.8	89.8 ± 9.1	90.9 ± 8.6	−1.1	−3.2; 1.0	0.319	0.12
W/H ratio	0.8 ± 0.1	0.8 ± 0.1	0.8 ± 0.1	0.1	0.5; 0.1	0.000	0.60

MS: male; FS: female; SR: Sit-and-reach test; PLSR: Passive straight leg raise; W/H ratio: Waist to hip ratio.

**Table 2:** Percentages (counts) of curvature classification, PA practice, leisure-time sedentary behaviors and SP and differences between sexes.

	General Sample 100% (*n* = 273)	MS 49.08% (*n* = 134)	FS 50.92% (*n* = 139)	r *	*p*
Thoracic curvature classification					
Hypokyphosis	8.8(24)	13.4 (18)	4.3(6)	0.241	0.000
Normal	71.4(195)	75.4(101)	67.6(94)		
Hyperkyphosis	19.8(54)	11.2(15)	28.1(39)		
Lumbar curvature classification					
Hypolordosis	2.2(6)	4.5(6)	0.0(0)	0.223	0.001
Normal	72.5(198)	78.4(105)	66.9(93)		
Hyperlordosis	25.3(69)	17.2(23)	33.1(46)		
PA practice					
No	15.7(43)	10.4(14)	20.9(29)	-0.143	0.018
Yes	84.2(230)	89.6(120)	79.1(110)		
Practice h PA/week					
0 h	16.7(43)	11.3(14)	21.6(29)	0.277	0.000
1-5 h	49.2(127)	41.9(52)	56.0(75)		
6-10 h	27.9(72)	36.3 (45)	20.1(27)		
+10 h	6.2(16)	10.4(13)	2.2(3)		
Leisure-time sedentary behaviors					
≤ 2 h	74.8(202)	70.9(95)	78.7(107)	0.090	0.141
>2 h	25.2(68)	29.1(39)	21.3(29)		
SP					
No	83.9(229)	83.6(112)	84.2(117)	0.008	0.894
Yes	16.1(44)	16.4(22)	15.8(22)		
CP					
No	97.7(255)	98.4(124)	97.0(131)	0.046	0.459
Yes	2.3(6)	1.6(2)	3.0(4)		
TSP					
No	94.2(246)	96.8(122)	91.8(124)	0.107	0.085
Yes	5.7(15)	3.2(4)	8.1(11)		
LBP					
No	89.7(234)	89.7(113)	89.6(121)	0.001	0.989
Yes	10.3(27)	10.3(13)	10.4(14)		

MS: male; FS: female; PA: physical activity; SP: spinal pain; CP: cervical pain; TSP: thoracic spine pain; LBP: low back pain; *: Cramer’s V or Coefficient contingence.

**Table 3:** Mean values (±SD) of curvatures, anthropometric variables and HRQL and differences between SP and sex.

	General Sample 100% (*n* = 273)			MS 49.08% (*n* = 134)			FS 50.92% (*n* = 139)		
	Without SP 83.88% (*n* = 229)	With SP 16.12% (*n* = 44)	*p*	Without SP 83.58% (*n* = 112)	With SP 16.42% (*n* = 22)	*p*	Without SP 84.17% (*n* = 117)	With SP 15.83% (*n* = 22)	*p*
Thoracic curve in standing (°)	35.3 ± 11.6	35.5 ± 10.4	0.931	31.6 ± 11.0	34.8 ± 9.6	0.204	38.8 ± 11.1	36.1 ± 11.4	0.291
Lumbar curve in standing (°)	−35.3 ± 7.2	−34.8 ± 7.5	0.661	−33.5 ± 7.1	−33.6 ± 8.6	0.883	−37.2 ± 6.7	−36.0 ± 6.3	0.434
Pelvic tilt in standing (°)	25.0 ± 7.4	25.6 ± 6.6	0.589	26.0 ± 8.2	25.9 ± 7.1	0.965	23.9 ± 6.5	25.2 ± 6.2	0.370
Pelvic tilt in SR (°)	−14.0 ± 13.0	−14.5 ± 13.7	0.753	−20.3 ± 10.0	−21.1 ± 7.3	0.729	−7.7 ± 12.5	−7.9 ± 15.5	0.936
Distance in SR (cm)	−8.6 ± 9.5	−9.3 ± 8.7	0.647	−12.6 ± 8.5	−14.3 ± 5.1	0.378	−4.8 ± 8.7	−4.3 ± 8.9	0.820
PSLR (°)	81.8 ± 14.2	82.1 ± 15.1	0.870	74.7 ± 9.4	75.6 ± 7.7	0.666	88.5 ± 14.8	88.7 ± 17.8	0.966
Age	13.1 ± 1.2	13.7 ± 1.3	0.011	13.0 ± 1.1	13.3 ± 1.2	0.398	13.2 ± 1.3	14.1 ± 1.25	0.006
Body mass (kg)	53.7 ± 12.0	56.7 ± 12.8	0.143	54.9 ± 12.8	58.7 ± 13.9	0.217	52.6 ± 11.1	54.7 ± 11.6	0.428
Height (cm)	161.0 ± 9.4	162.9 ± 9.2	0.231	162.8 ± 10.9	165.1 ± 10.6	0.381	159.3 ± 7.4	160.7 ± 7.3	0.412
BMI (kg/m^2^)	20.6 ± 3.7	21.3 ± 4.4	0.288	20.6 ± 3.8	21.4 ± 4.1	0.386	20.6 ± 3.7	21.2 ± 4.7	0.529
Waist girth (cm)	72.3 ± 9.8	72.6 ± 10.5	0.843	74.9 ± 10.3	74.9 ± 11.5	0.986	69.8 ± 8.6	70.3 ± 9.0	0.806
Hip girth (cm)	90.1 ± 8.7	91.3 ± 9.2	0.424	89.5 ± 9.0	91.3 ± 9.3	0.408	90.8 ± 8.5	91.4 ± 9.2	0.768
W/H ratio	0.8 ± 0.1	0.8 ± 0.1	0.492	0.8 ± 0.1	0.8 ± 0.1	0.237	0.8 ± 0.1	0.8 ± 0.1	0.997
Health related quality of life (HRQL)									
Physical well being	4.0 ± 0.6	3.6 ± 0.8	0.001	4.1 ± 0.6	3.9 ± 0.6	0.197	3.9 ± 0.6	3.4 ± 0.8	0.001
Psychological well being	1.9 ± 0.5	1.7 ± 0.7	0.050	4.1 ± 0.6	3.9 ± 0.6	0.261	3.9 ± 0.6	3.4 ± 0.8	0.038
Autonomy and relationship with parents	4.4 ± 0.6	4.1 ± 0.6	0.002	4.4 ± 0.6	4.1 ± 0.6	0.075	4.4 ± 0.6	4.0 ± 0.6	0.009
Social and peer support	4.7 ± 0.5	4.5 ± 0.6	0.099	4.6 ± 0.6	4.5 ± 0.6	0.778	4.8 ± 0.5	4.5 ± 0.6	0.023

MS: male; FS: female; SR: Sit-and-reach test; PLSR: Passive straight leg raise; W/H ratio: Waist to hip ratio.

**Table 4:** Percentages (counts) of curvatures classification, PA and leisure-time sedentary behaviors and differences between SP and sex.

	General Sample 100% (*n* = 273)			MS 49.08% (*n* = 134)			FS 50.92% (*n* = 139)		
	Without SP 83.88% (*n* = 229)	With SP 16.12% (*n* = 44)	*p*	Without SP 83.58% (*n* = 112)	With SP 16.42% (*n* = 22)	*p*	Without SP 84.17% (*n* = 117)	With SP 15.83% (*n* = 22)	*p*
Thoracic curvature classification									
Hypokyphosis	9.2(21)	6.8(3)	0.880	15.2(17)	4.6(1)	0.258	3.4(4)	9.1(2)	0.439
Normal	71.2(163)	72.7(32)		75.0(84)	77.3(17)		67.5(79)	68.2(15)	
Hyperkyphosis	11.6(45)	20.4(9)		9.8(11)	18.2(4)		29.1(34)	22.7(5)	
Lumbar curvature classification									
Hypolordosis	1.7(4)	4.6(2)	0.484	3.6(4)	9.1(2)	0.170	0.0(0)	0.0(0)	0.105
Normal	72.5(166)	72.7(32)		81.2(91)	63.6(14)		64.1(75)	81.8(18)	
Hyperlordosis	25.8(59)	22.7(10)		15.2(17)	27.3(6)		35.9(42)	18.2(4)	
PA practice									
No	14.8(34)	20.4(9)	0.350	10.7(12)	9.1(2)	0.820	18.8(22)	31.8(7)	0.168
Yes	85.1(195)	79.5(35)		89.3(100)	90.9(20)		81.2(95)	68.2(15)	
Practice h PA/week									
0 h	15.6(34)	22.5(9)	0.741	11.4(12)	10.5(2)	0.684	19.5(22)	33.3(7)	0.292
1–5 h	50.0(109)	45.0(18)		42.9(45)	36.8(7)		56.6(64)	52.4(11)	
6–10 h	28.0(61)	27.5(11)		34.3(36)	47.4(9)		22.1(25)	9.5(2)	
+10 h	6.4(14)	5.0(2)		11.4(12)	5.3(1)		1.8(2)	4.8(1)	
Leisure-time sedentary behaviors									
≤ 2 h	77.9(176)	59.1(26)	0.009	72.3(81)	63.6(14)	0.412	83.3(95)	54.6(12)	0.003
>2 h	22.1(50)	40.9(18)		27.7(31)	36.4(8)		16.7(19)	45.4(10)	

MS: male; FS: female; PA: physical activity; SP: Spinal pain.

**Table 5:** Percentages (counts) of sedentary and active behaviors variable and differences between SP and sex.

	General Sample 100% (*n* = 273)			MS 49.08% (*n* = 134)			FS 50.92% (*n* = 139)		
	Without SP 83.88% (*n* = 229)	With SP 16.12% (*n* = 44)	*p*	Without SP 83.58% (*n* = 112)	With SP 16.42% (*n* = 22)	*p*	Without SP 84.17% (*n* = 117)	With SP 15.83% (*n* = 22)	*p*
Not sedentary and active	86.59(71)	13.41(11)	0.014	84(42)	16(8)	0.585	90.63(29)	9.38(3)	0.011
Not sedentary and not active	88.24(105)	11.76(14)		88.64(39)	11.36(5)		88(66)	12(9)	
Sedentary and active	87.50(14)	12.50(2)		85.71(12)	14.29(2)		100(2)	0(0)	
Sedentary and Not active	69.23(36)	30.77(16)		76(19)	24(6)		62.96(17)	37.04(10)	

MS: male; FS: female; PA: physical activity; SP: Spinal pain.

**Table 6:** Stepwise multiple logistic regression analysis of the predictor variable of SP according to age and leisure time sedentary behaviors.

	Predictor Variable of SP	Adjusted Odd Ratio	SE	95% Confidence Interval		*p*
				Lower	Upper	
Model 1	Age	1.42	0.131	1.10	1.84	0.007
Model 2	Age	1.35	0.134	1.04	1.76	0.023
	Leisure-time sedentary behaviors ± 2 h	2.10	0.355	1.04	4.21	0.037
